# Epidemiology of Respiratory Pathogens in Children with Severe Acute Respiratory Infection and Impact of the Multiplex PCR Film Array Respiratory Panel: A 2-Year Study

**DOI:** 10.1155/2021/2276261

**Published:** 2021-12-31

**Authors:** Asmae Lamrani Hanchi, Morad Guennouni, Meriem Rachidi, Toufik Benhoumich, Hind Bennani, Mounir Bourrous, Fadl Mrabih Rabou Maoulainine, Said Younous, Mohamed Bouskraoui, Nabila Soraa

**Affiliations:** ^1^Faculty of Medicine and Pharmacy, Cadi Ayyad University, Laboratory of Microbiology, University Hospital Mohamed VI, Marrakech, Morocco; ^2^Hassan First University of Settat, Higher Institute of Health Sciences of Settat, Laboratory of Health Sciences and Technologies, Settat, Morocco; ^3^Faculty of Medicine and Pharmacy of Marrakech, Cadi Ayyad University, Paediatric Emergency Department, University Hospital Mohamed VI, Marrakech, Morocco; ^4^Faculty of Medicine and Pharmacy of Marrakech, Cadi Ayyad University, Neonatal Intensive Care Unit Department, University Hospital Mohamed VI, Marrakech, Morocco; ^5^Faculty of Medicine and Pharmacy of Marrakech, Cadi Ayyad University, Paediatric Intensive Care Unit, University Hospital Mohamed VI, Marrakech, Morocco; ^6^Faculty of Medicine and Pharmacy of Marrakech, Cadi Ayyad University, Paediatric Department, University Hospital Mohamed VI, Marrakech, Morocco

## Abstract

Sever acute respiratory infections (SARIs) are a public health issue that are common in children and are associated with an important morbidity and mortality rate worldwide. Although SARI are mainly caused by viruses, they are still a cause of antibiotic overuse. The use of molecular methods especially real-time multiplex PCR allowed to detect a wide range of respiratory viruses and their subtype as well as some atypical bacteria. The aim of this study was to investigate the epidemiology of respiratory pathogens detected in children admitted with SARI and to highlight the role of real-time multiplex PCR in the rapid diagnosis of viral and bacterial SARI. This work is a descriptive observational study from January 2018 to December 2019 including nasopharyngeal secretions collected from 534 children hospitalised in paediatric department. The detection of respiratory viruses and bacteria was performed by the FilmArray® Respiratory Panel. A total of 387 (72.5%) children were tested positive for at least one respiratory pathogen, and 23.3% of them were coinfected with more than one pathogen. Viral aetiology was found in 91.2% (*n* = 340). The most common viruses detected were HRV (*n* = 201) and RSV (*n* = 124), followed by PIV (*n* = 35) influenza A (*n* = 29) and human metapneumovirus (*n* = 27). Bacteria was found in 8.8% (*n* = 47), and Bordetella pertussis was the most detected. Respiratory syncytial virus and Bordetella pertussis were significantly higher in infants less than 6 months old. The detection of RSV and influenza A presented a pic in winter, and HMPV was statistically significant in spring (*p* < 0.01). This study described the epidemiology of respiratory pathogens involved in severe respiratory infections in children that were affected by several factors such as season and age group. It also highlighted the importance of multiplex PCR in confirming viral origin, thus avoiding irrational prescription of antibiotics in paediatric settings.

## 1. Introduction

Severe acute respiratory infections (SARIs) are a public health issue. They are the main infectious disease in children who are particularly vulnerable [1, 2]. SARIs are the most common cause of hospitalisation during childhood and are associated with an important mortality rate worldwide [3, 4]. Indeed, they are the third leading cause of death in the world [5]. SARIs represent a challenge for healthcare systems, especially in low- and middle-income countries. Infant and child mortality is lower in high-income countries. It can be explained by several factors such as vaccination, easy access to treatment, nutrition, and improved sanitary conditions [6]. In Morocco, there has been a significant reduction in the infant mortality rate over the past decades, due to the progress in strengthening vaccination programmes and control of infectious diseases, including acute respiratory infections [7, 8]. Furthermore, understanding the epidemiology and aetiology of acute respiratory infections in hospitalised children is essential for the development of a strategy for the rapid and optimal management of these infections, as well as the implementation of means to prevent and control them [9].

SARI are due to viruses in the majority of cases [10]. However, atypical bacteria can also be involved [2, 6]. Despite this, respiratory infections are a major cause of antibiotic overuse [11]. Antibiotics are not only ineffective but also expose children to adverse effects and contribute to the emergence and spread of multidrug-resistant bacteria and increased economic costs associated with infections [9].

The use of molecular methods, especially real-time multiplex polymerase chain reaction (mPCR), has considerably improved the detection of respiratory pathogens [12]. The mPCR allowed to detect a wide range of respiratory viruses and their subtype as well as some atypical bacteria with high sensitivity and specificity [13, 14]. The molecular diagnosis using a syndromic approach for the detection of respiratory pathogens is being used significantly more in paediatric hospitals and allows a rapid distinction between viral and bacterial infections especially by the FilmArray® Respiratory Panel (FA-RP) [9]. It facilitates the management of patients with SARI and guides the clinical decision. Moreover, it helps to limit their spread, reduces antibiotics overuse, and guides the development of new vaccines [9, 15].

In Morocco, to our knowledge, limited studies have been conducted to identify epidemiology and aetiology profile of acute respiratory tract infections in paediatric patients. The aim of this study is to investigate the epidemiology of respiratory pathogens detected in children admitted with SARI and to highlight the role of real-time multiplex PCR in the rapid diagnosis of viral and bacterial acute respiratory tract infections.

## 2. Patients and Methods

### 2.1. Patients

This study was a descriptive observational and retrospective analysis using data from a microbiology laboratory. This study covers a period of 24 months from January 2018 to December 2019 including nasopharyngeal secretions and respiratory specimens collected from 534 children younger than fourteen years old hospitalised in the University Hospital Med VI of Marrakech. Our paediatric hospital is the only tertiary hospital covering the region of Marrakech, which receives referred children from the south of Morocco.

We have included in our study children under 14 years old with severe bronchiolitis, respiratory distress, pneumonia, influenza-like illness in immunocompromised children, and clinical suspicion of pertussis that required a hospitalisation in paediatric departments, while we excluded children with noninfectious or chronic respiratory disease from this study. The diagnosis is based on clinical symptoms and clinical examination such as severe cough, tachypnoea ≥60 c/min, wheezing, signs of respiratory struggle and SpO2 ≤90%, and abnormal pulmonary auscultation, as well as radiological evidence of SARI where a radiological assessment was required. Nasopharyngeal secretions were collected from children enrolled in this study, using a flocked nasopharyngeal swab (NPS), on the basis of the standard technique, and then immersed in a viral transport medium. Distal respiratory specimens (protected distal brush or bronchoalveolar lavage) were taken from intubated children hospitalised in the paediatric intensive care unit [10, 16]. The samples are processed as soon as they are received in the microbiology laboratory for the etiological diagnosis. The results are provided on average in two hours.

### 2.2. Methods

The detection of respiratory viruses and bacteria was performed by the filmArray® instrument (bioMérieux, France) with the FilmArray® Respiratory Panel (FA-RP), which simultaneously detects viruses and bacteria in less than an hour. In brief, the FilmArray pouch was prepared with 1 mL of hydration solution and 300 *μ*l of NPS and sample buffer, according to the manufacturer's protocol. Then, the pouch was integrated into the FilmArray instrument, and a preprogramed run was started. The FilmArray is a multiplex PCR system that integrates sample extraction, amplification, detection, and analysis. The following respiratory viruses detected are human adenovirus (AdV), human coronavirus (CoV) 229E, NL63, HKU1 and OC43, human metapneumovirus (HMPV), human rhinovirus (HRV), human enterovirus (EnV), influenza virus A (Inf A), A/H1, A/H1-2009, and A/H3 and B(Inf B), parainfluenza virus 1, 2, 3, and 4 (PIV), and respiratory syncytial virus (RSV). The bacteria detected in this respiratory panel are Bordetella pertussis (Bp), Chlamydophila pneumoniae (Chp), and Mycoplasma pneumonia (Mp) [17, 18].

### 2.3. Ethics, Authorisation, and Approval

The study focuses on children. Hence, the anonymity of each patient was considered according to recommendations of the Declaration of Helsinki. The study was approved by the ethics committee of the Faculty of Medicine and Pharmacy of Marrakech (N° 22/2021).

### 2.4. Statistical Analysis

All statistical analysis was performed using SPSS software (version 23.0; SPSS, Inc., Chicago, IL, USA) and Microsoft Excel (Microsoft Corporation, Washington, USA). Statistical comparisons were performed using the chi-square test. A probability (*p*) value less than 0.05 was considered statistically significant.

## 3. Results

### 3.1. Paediatric Patient Demographics and Clinical Characteristics

During the study period, a total of 534 specimens from paediatric inpatients were tested using FilmArray RP. The children included in this study were divided into five groups according to age: 0 to 6 months (M), 6 M to 1 year (Y), 1 to 2 Y, 2 to 5 Y, and more than 5 Y old. More than half of the patients (*n* = 292, 54.7%) were less than 6 months old. The prevalence of SARI was higher among males (*n* = 310, 58.1%).

**Figure 1:**
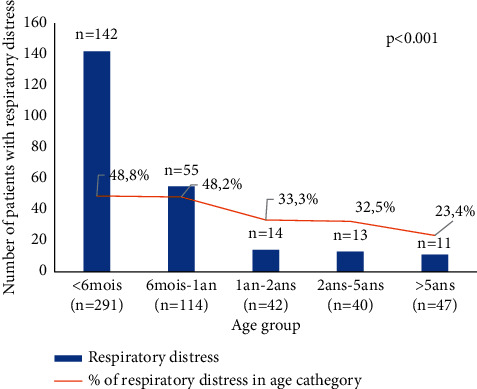
Prevalence of respiratory distress according to age group.

Among the inpatients, 59% were hospitalised in the paediatric department, 18% in the paediatric emergency department, and 13% and 8.6% in the intensive care unit and the neonatology intensive care unit, respectively. Overall, respiratory distress was the most common reason for hospitalisation mainly in children less than 1 year old (*p* < 0.05) (Figure 1). The admission of children with SARI was higher in winter (*n* = 200, 37.5%). A summary of the demographics and clinical characteristics of patients is shown in Table 1.

### 3.2. Distribution of Respiratory Pathogens

**Figure 2:**
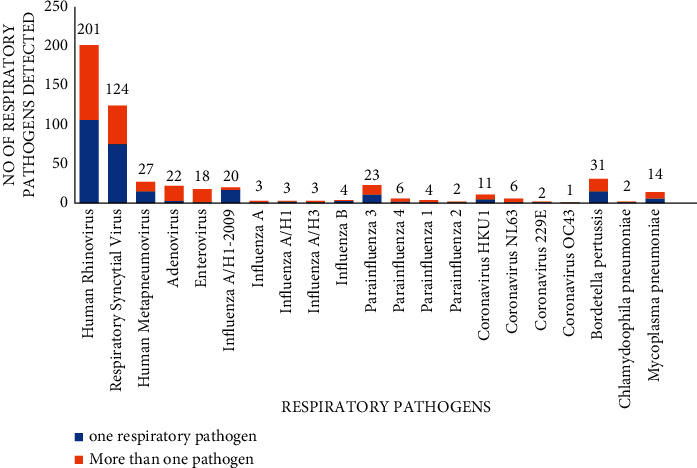
Distribution of respiratory pathogens in children.

A total of 387 (72.5%) of the children were tested positive for at least one respiratory pathogen, and 23.3% of these were coinfected with more than one pathogen. The rate of positivity in boys was similar to girls (*n* = 225/310, 72.6%; *n* = 162/224, 72.3%; *p*=0.947,). Viral aetiology was predominant in SARI and found in 91.2% (*n* = 340) of cases. The most frequent viruses detected were HRV (*n* = 201) and RSV (*n* = 124), followed by PIV (*n* = 35), influenza A (*n* = 29), and human metapneumovirus (*n* = 27). However, bacteria was found in 8.8% (*n* = 47), and Bordetella pertussis was the most detected (*n* = 31) (Figure 2).

### 3.3. Age Distribution of Severe Acute Respiratory Infections

The difference in the positivity rate according to age group was statistically significant (*p* < 0.001). It was significantly lower in children more than 5 years old compared to those less than 5 (Table 2).  Table 3 summarises the distribution of the respiratory pathogens in each age group. HRV was the most common respiratory pathogen in all age groups. Respiratory syncytial virus and Bordetella pertussis were significantly higher in infants less than 6 months old (*p* < 0.0001 and *p* < 0.05, respectively). Parainfluenza was found especially in infants between 6 months and 1 year old (*p*=0.014), and influenza A was dominant in children between 2 and 5 years old (*p* < 0.0001).

### 3.4. Co-Infection

As shown in Table 2, the prevalence of co-infections was significantly higher in children less than 2 years old than in those more than 2 (*p* < 0.005) and decreased with age. The most frequent respiratory pathogen involved in co-infection was HRV (Table 4). Among 119 patients with co-infection, two, three, and four pathogens were detected in 99, 19, and one case, respectively.

### 3.5. Seasonality

**Figure 3:**
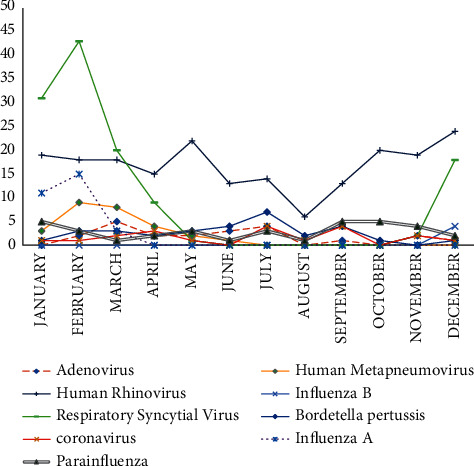
Monthly distribution of respiratory pathogens.

The seasonality of the detection rate of respiratory pathogens was statistically significant (*p* < 0.001). It was the highest in winter (*n* = 165, 82.5%), followed by spring (*n* = 104, 68.9%), autumn (*n* = 69, 66.3%), and summer (*n* = 49, 62%). HRV was detected throughout the year but statistically higher in autumn. The same trend was observed for PIV. The detection of RSV presented a pic in winter (*p* < 0.0001), especially in January and February. This is also the case for the distribution of influenza A. However, the detection of HMPV in spring was statistically significant particularly in March. As for Bordetella pertussis, it was significantly higher in summer (*p* < 0.0001) **(**Figure 3) and (Table 5).

## 4. Discussion

Through this study, we have described and analysed the epidemiology of respiratory pathogens involved in acute respiratory infections in children. This study confirmed the high prevalence of viral aetiology in paediatric patients and justified the usefulness of the implementation of molecular techniques such as multiplex PCR in the diagnosis of these infections. Inappropriate use of antibiotics for viral infections is an important factor in increasing multidrug resistance. Therefore, laboratory tests should be included to determine the aetiology and, thus, to provide the appropriate treatment [16]. SARI infections are frequent especially in children [6]. In the first year of life, infants are particularly vulnerable to respiratory infections according to Esposito et al. [2]. Our study confirmed this vulnerability showing that 76.5% (406/534) of children admitted with SARI were less than one year old. Similar findings have been noted by studies conducted in China [1, 4, 18] and in the Netherlands [19]. In contrast to this finding, few studies such as that conducted by Lei et al. [20] have reported that SARI were more common in patients aged one to three years (52%). Furthermore, almost all infants under two years old developed at least a respiratory infection due to RSV and/or RV [21]. This susceptibility to these infections can be explained by some features of the infant's immune response. The latter is characterised early by the deficiency of immunological memory and a biased tolerogenic immune response (T cells including Tregs and Th2), whereas Th1 immunity is limited and associated with disease severity [22].

In this study, male dominance in children with SARI was found (58.1%), confirming the results of previous studies [4, 19, 23, 24]. According to McClelland and Smith [25], this dominance can be explained by an interaction between gender-specific immune responses and immune responses to specific microbes. It has been demonstrated that male children are more susceptible to severe illness when infected with RSV due to immune response, airway mechanics, and smaller airway.

Respiratory distress related to SARI was found in 44% of our patients, indicating the severity of the clinical features. This can be explained by the young age of our study population, which was mainly less than one year, and whose respiratory distress was significantly correlated (*p* < 0.05).

In this research, it was noticeable that the hospitalisations were particularly common in the coldest season. The same trend has been observed in our country in a previous study carried out by Jroundi et al. [24]. Moreover, respiratory infections, in particular viral bronchiolitis in infants, evolve in epidemics during the winter period [26]. However, it depends on geographical localisation, climate, and the respiratory pathogen involved in this infection [1].

Among the 534 patients enrolled in this study, the overall prevalence of respiratory pathogens was 72.5% (*n* = 387) and the proportion of co-infections was 23.3%. Our positivity rate is within the range found in the literature, which varied between 51.4% and 92% [12, 19]. Multiple viruses or co-infection bacteria and virus were found in 15–33.5% [6, 12, 20]. This disparity in the prevalence of detection of respiratory pathogens may be due to the difference in the inclusion criteria of the cases in the different studies, the geographical location, the seasons, and the panel used for the molecular diagnosis [1]. The high rate of positivity in our study can be explained by the use of larger panel of respiratory pathogens as well as more sensitive molecular techniques, which detect more than 20 respiratory pathogens simultaneously.

Differences in the positivity rate and distribution of respiratory pathogens according to age groups were noticed in this research. It was found that children less than five years old had the highest positivity rate. A high prevalence among children between 6 and 36 months old has been reported by Visseaux et al. and Wen et al. [4, 12]. The same results were reported by Lei et al. [20] who found the lowest prevalence of detection of respiratory pathogens in children over six years old. The co-infection decreased with age and was significantly higher in the children less than 2 years old than in those more than 2 (*p* < 0.005) through this study. Wen et al. [4] found a prevalence of co-infection statistically significant in infants (<1 year old) and toddler (1–3 years old).

In line with other studies [4, 12, 24, 27, 28], viruses were the most respiratory pathogen detected in our research. This underlines the usefulness of mPCR in the etiological diagnosis of respiratory infections in children due to their high sensitivity, specificity, and rapidity, [13] especially when it is difficult to identify the aetiologic pathogen on the basis of symptoms only. Indeed, respiratory infections are characterised by low specificity of clinical assessment [4]. However, the need to distinguish between a bacterial and a viral infection is crucial, as the management is totally different. The syndromic approach allows the detection of one or multiple respiratory pathogens including viruses and atypical bacteria simultaneously in less than one hour. It enables the determination of the etiological diagnosis of an acute respiratory infection, providing timely decisions on hospital admission, appropriate treatment, and thus the infection control and the reduction of the social and economic problems related to paediatric respiratory diseases [2, 13]. In fact, classical microbiology techniques can detect bacteria, but cannot allow the identification of respiratory viruses. Viral culture was previously the gold standard for the diagnosis of viral infections, but it is not adapted for diagnosis in an emergency context and has been replaced by multiplex PCR [16, 26].

Regarding the profile of respiratory pathogen detected in the present study, the most common viruses were HRV, followed by RSV (*n* = 124), PIV (*n* = 35), influenza A (*n* = 29), and human metapneumovirus (*n* = 27). Nevertheless, ADV and CoV were only detected in 4.1% and 3.7% of cases, respectively. These results are comparable to those reported by several studies [19, 24]. It was noticeable that HRV was the most detected respiratory pathogen in studies conducted in our country and other ones [1, 4, 9, 18, 19, 20, 24, 29]. Lei et al. [20] have noted in their study that HRV was responsible for both upper and lower respiratory infections. Furthermore, HRV has been found to be involved in respiratory infections especially in prematurely born infants due to their functional and genetic predisposition [30] as well as the triggering of asthma attacks [22, 31, 32]. Despite the fact that HRV is increasingly involved in respiratory infections in all age groups, it is still the subject of much debate [3, 20]. Moreover, viruses can usually colonise the respiratory tract asymptomatically for several weeks after a cured infection or during the asymptomatic incubation period [2]. In fact, according to the study by Principi et al. [33], the most commonly found virus in asymptomatic patients was the HRV, whose detection decreases with age [34]. In a prospective study of 20 healthy infants during the first year of live, the authors concluded that the detection of HRV in the airways in infants was very heterogeneous and dynamic, and about 50% of the HRV infections in infants less than one year have no respiratory symptoms [35]. In accordance with these findings, RSV, IFV, PIV, MPV, and HRV were significantly more prevalent in paediatric inpatients with SARI than in asymptomatic children according to a systematic review and meta-analysis [3]. Nevertheless, there was less evidence for the involvement of HRV in respiratory infections in children than for RSV, PIV, MPV [36]. However, Self et al. suggest, in their study, that HRV plays an etiological role in acute respiratory infection when detected in a symptomatic patient [34]. Similarly, several recent multicentre studies have demonstrated an increasing evidence suggesting a causal role for rhinovirus in the aetiology of SARI, particularly in young children [37–40]. These findings, lending further evidence of the pathogenicity of this virus particularly in young children, are consistent with our results, where all children who tested positive for HRV had SARI. About its distribution related to the seasons, many studies have noted its detection throughout the year [20, 28, 41]. The same trend was found in our study, with a statistically significant detection in autumn (*p* < 0.01). This predominance in autumn has also been reported in China by Zhao et al. [1].

In contrast to HRV, RSV was more common in children under six months, especially during the coldest season. A significant correlation between the occurrence of RSV respiratory infection and the cold and rainy season was observed by Jain et al. [28]. This finding has been supported by previous studies [1, 4, 19, 20, 23, 27]. In a systematic review and meta-analysis conducted by Stein et al. [23], preterm infants were three times more likely to develop an SARI due to RSV in the first year of life with a risk of severe episode and hospitalisation. The authors concluded that RSV respiratory infection is an important cause of hospitalisation and a significant factor in paediatric mortality [23]. Therefore, it is imperative to establish measures to manage and control RSV disease during the coldest season. Indeed, respiratory viruses including RSV are responsible for nosocomial infections, particularly in children. Therefore, it is also important to limit its spread by isolating hospitalised children and by reinforcing hygiene measures among health personnel since the transmission of RSV is mainly by droplets and contact [42].

It was noted that all PIV subtypes were found in 6.6% of the cases, with the predominant subtype being Piv 3 (23/35), among infants aged 6 to 12 months especially in autumn with statistical significance (*p* < 0.05). Similar findings have been noted in other studies [1, 4]. As for the Inf A, it was found especially in older children (2 to 5 years old) in the cold season.

In our study, HMP accounted for 5.1% of all respiratory pathogens detected. This result is comparable with other studies [43]. It was found in all age groups with no significant difference, especially in spring in consent with other studies (*p* < 0.05) [4, 20]. Concerning ADV, it was less prevalent in this study in contrast to others [1, 24]. It was found more in spring and summer, and its detection decreases with age in agreement with other studies [44].

Multiplex RT-PCR allows the detection of co-infection and enables us to estimate considerably the prevalence of multiple viral infections. It was statically more significant in children less than two years in our study (*p* < 0.001) as demonstrated in other studies [45]. According to Yen et al. [45], the multiple infections were not associated with a worse outcome such as duration of fever, respiratory symptoms, and hospitalisation. HRV was the most recurrent respiratory pathogen in multiple infections. It was most commonly associated with RSV, followed by ADV, B. pertussis, and PIV.

Although SARI are essentially caused by viruses, atypical bacteria including B. pertussiss, M. pneumoniae, and C. pneumoniae should also be suspected as they require a specific treatment [18, 24]. In the present study, atypical bacteria were found in 8.8% of cases, with B pertussis being the most common particularly in infants under 6 months during the summer.

The description and the analysis of the epidemiological profile of the respiratory pathogens in the present study were carried out through the molecular diagnosis using multiplex RT-PCR. The implementation of this rapid molecular test for the detection of respiratory pathogens improved the diagnostic efficiency of the laboratory since in our study, more than 90% of the SARI were viral, and has considerably reduced the lead time and the time required to provide results. Indeed, the FilmArray RP platform is easy to use and requires minimal handling time [17]. In the study conducted by Lee et al. [46], the authors concluded that the adoption of a molecular respiratory panel with a short turnaround time provides significant benefit and improves decision-making for patient management by reducing antibiotic use and chest X-ray prescription, as well as increasing the timely use of oseltamivir and finally decreasing the length of inpatients stay. Furthermore, Brendish et al. [47] found in their study that the rapid turnaround time was strongly correlated with earlier hospital discharge and early antibiotic discontinuation. They suggest that there is an early “window of opportunity” for the results of molecular diagnostic tests to change patient management and support the turnaround time as a crucial determinant of medical decision-making. However, the cost-effectiveness of a molecular diagnosis for respiratory pathogens in paediatric patients remains to be determined. Despite the fact that the diagnostic strategy for SARI based on multiplex PCR with a syndromic approach is costly, it is likely to reduce the overall cost of care as it significantly reduces the length of hospital stay and antibiotic use [47].

### 4.1. Strengths and Limitations

Our study presents some strengths and limitations. This is a large series of patients and was conducted over a period of 2 years. The molecular technique used for the etiological diagnosis of SARI has a high sensitivity and specificity with a rapid turnaround time. However, this is a study with no information on the management, clinical course, and assessment of the impact of mPCR on the outcomes of our paediatric patients. Further studies need to be conducted to better evaluate the impact of mPCR in children outcomes and linked this with clinical data as they have important implications for disease control policy for our country.

## 5. Conclusion

This study described and analysed the epidemiology of respiratory pathogens involved in severe acute respiratory infections in children, which are characterised by their diversity and their different distribution according to season and age group. This study also highlighted the importance of mPCR in confirming the viral origin and thus avoiding irrational prescription of antibiotics in paediatric settings.

## Figures and Tables

**Table 1:** Paediatric patient demographics and clinical characteristics (*n* = 534).

Gender	
Male	**310 (58.1)**
Female	**224 (41.9)**

Age in months (mean/median)	**17.6 (4)**
Age range	
<6 months	**292 (54.7)**
6 months to 1 year	**114 (21.3)**
1 to 2 years	**42 (7.9)**
2 to 5 years	**40 (7.5)**
>5 years	**46 (8.7)**

Clinical diagnosis	
Bronchiolitis	**180 (33.7)**
Severe bronchiolitis with respiratory distress	**235 (44)**
Pneumonia	**83 (15.5)**
Pertussis	**31 (5.8)**
Influenza in immunocompromised children	**34 (6.3)**
Other	**20 (3.7)**

Season of admission	
Autumn	**104 (19.5)**
Winter	**200 (37.5)**
Spring	**151 (28.3)**
Summer	**79 (14.8)**

**Table 2:** Prevalence of respiratory pathogens in the five age groups (*n* = 534).

	Total no. of samples	Total no. of negative samples (%)	Total no. of positive samples (%)	Total no. of co-infections samples
[<6 months]	292	71 (24.4)	221 (**75.7**)	69 (23.6)
[6 months-1 years]	114	32 (28.1)	82 (**71.9**)	28 (24.6)
[1-2Years]	42	11 (26.2)	31 (**73.8**)	13 (31)
[2–5 years]	40	8 (20)	32 (**80**)	3 (7.5)
[>5 years]	46	25 (53.2)	21 (**45.6**)^*∗∗*^	6 (13)
Total	534	147 (27.5)	387 (**72.5**)^*∗*^	119 (22.3)^*∗*^

(i) ^*∗*^All group *p* < 0.001. (ii) ^*∗∗*^ compared with <5 y, *p* < 0.0001.

**Table 3:** Distribution of respiratory pathogens in each age group.

	[< 6 months] *n* = 292 *n* (%)	[6 months –1 Year] *N* = 114 *n* (%)	[1–2 Years] *N* = 42 *n* (%)	[2–5 years] *N* = 40 *n* (%)	[>5 years] *N* = 46 *n* (%)	Total *N* = 534 *n* (%)	*p* value
Human rhinovirus	116 (39.9)	42 (36.8)	19 (45.2)	11 (27.5)	13 (27.7)	201 (37.6)	0.274
Respiratory syncytial virus	94 (32.3)	19 (16.7)	5 (11.9)	4 (10)	2 (4.3)	124 (23.2)	**0.000001**
Parainfluenza	11 (3.8)	15 (13.2)	2 (4.8)	3 (7.5)	4 (8.5)	35 (6.6)	0.014
Influenza A	6 (2.1)	8 (7)	5 (11.9)	8 (20)	2 (4.3)	29 (5.4)	**0.000019**
Human metapneumovirus	12 (4.1)	9 (7.9)	3 (7.1)	3 (7.5)	0	27 (5.1)	0.213
Adenovirus	8 (2.7)	9 (7.9)	4 (9.5)	0	1 (2.1)	22 (4.1)	**0.028**
Coronavirus	12 (4.1)	4 (3.5)	1 (2.4)	2 (5)	1 (2.1)	20 (3.7)	0.92
Enterovirus	11 (3.8)	4 (3.5)	2 (4.8)	0	1 (2.1)	18 (3.4)	0.731
Influenza B	2 (0.7)	1 (0.9)	0	1 (2.5)	0	4 (0.7)	0.670
Bordetella pertussis	26 (8.9)	1 (0.9)	2 (4.8)	1 (2.5)	1 (2.1)	31 (5.8)	**0.015**
Chlamydophila pneumoniae	1 (0.3)	1 (0.9)	0	0	0	2 (0.4)	0.867
Mycoplasma pneumoniae	3 (1)	3 (2.6)	3 (7.1)	2 (5)	3 (6.4)	14 (2.6)	0.045

PIV includes piv1, piv2, piv3, and piv4. CoV includes CoV HKU1, CoV NL63, CoV OC43, and CoV 229E. Influenza A includes Inf A/H1-2009, Inf A/H1, and Inf A/H3.

**Table 4:** Prevalence of co-infection of respiratory pathogens in children.

	HRV	RSV	PIV	Inf A	HMPV	AdV	CoV	EnV	Inf B	Bp	Ch p
VRS	38										
PIV	13	2									
Inf A	2	5	1								
HMPV	4	4	3	1							
AdV	14	5	0	0	1						
CoV	8	0	0	0	1	3					
EnV	15	3	2	0	0	2	2				
Inf B	0	1	0	0	0	0	0	0			
Bordetella pertussi	14	0	0	0	0	1	5	0	0		
Ch p	1	0	0	0	0	0	0	0	0	0	
M p	5	1	2	1	0	2	0	0	0	0	0

**Table 5:** Prevalence of respiratory pathogens in seasons.

	Autumn *n* = 104 (%)	Winter *n* = 200 (%)	Spring *n* = 151(%)	Summer *n* = 79 (%)	Total *N* = 534	*p* value
Human rhinovirus	52 (50)	61 (30.5)	55 (36.4)	33 (41.8)	201	0.008
Respiratory syncytial virus	2 (1.9)	92 (46)	30 (19.9)	0	124	<0.0001
Parainfluenza	14 (13.5)	10 (5)	6 (4)	5 (6.3)		0.014
Influenza A	0	26 (13)	3 (2)	0	29	<0.0001
Human metapneumovirus	0	12 (6)	14 (9.3)	1 (1.3)	27	0.003
Adenovirus	3 (2.9)	3 (1.5)	9 (6)	7 (8.9)	22	0.022
Coronavirus	6 (5.8)	3 (1.5)	6 (4)	5 (6.3)	20	0.14
Enterovirus	7 (6.5)	4 (2)	5 (3.3)	2 (2.5)	18	0.17
Influenza B	0	4 (2)	0	0	4	0.08
Bordetella pertussis	5 (4.8)	5 (2.5)	8 (5.3)	13 (41.9)	31	0.00012
Chlamydophila pneumoniae	2 (1.9)	0	0	0	2	0.04
Mycoplasma pneumoniae	4 (3.8)	2 (1)	5 (3.3)	3 (3.8)	14	0.337

PIV includes piv1, piv2, piv3, and piv4. CoV includes CoV HKU1, CoV NL63, CoV OC43, and CoV 229E. Influenza A includes Inf A/H1-2009, Inf A/H1, and Inf A/H3. Autumn: September-October-November; winter: December-January-February; spring: March-April-May; summer: June-July-August.

## Data Availability

The Excel and SPSS file data used to support the findings of this study are available from the corresponding author upon request.

## Abbreviations

SARI: Sever acute respiratory infections
PCR: Polymerase chain reaction
mPCR: Multiplex PCR
HRV: Human rhinovirus
RSV: Respiratory syncytial virus
PIV: Parainfluenza virus
Inf A: Influenzae A
HMPV: Human Metapneumovirus
NPS: Nasopharyngeal secretions
M: Months
Y: Year
RP: Respiratory panel
AdV: Human adenovirus
Chp: Chlamydophila pneumoniae
Bp: Bordetella pertussis
Mp: Mycoplasma pneumonia
EnV: Human enterovirus
CoV: Coronavirus
Th1: Lymphocyte T helper 1
Th2: Lymphocyte T helper 2
Treg: Lymphocyte T regulator
RT-PCR: Real-time PCR.
